# Refining Complete Dentures: Optimizing Results with Enhanced Phonetics and Esthetics

**DOI:** 10.7759/cureus.62938

**Published:** 2024-06-23

**Authors:** Rucha Chiddarwar, Anjali Bhoyar, Surekha A Dubey, Mithilesh M Dhamande, Sheetal R Khubchandani

**Affiliations:** 1 Prosthodontics, Sharad Pawar Dental College and Hospital, Datta Meghe Institute of Higher Education and Research, Sawangi, Wardha, IND

**Keywords:** characterization, staining, palatogram, neutral zone technique, ridge resorption

## Abstract

Tooth loss and subsequent complete denture rehabilitation can profoundly affect a patient’s psychological and social well-being. Dentures play an important role in helping individuals regain a sense of normalcy as well as facilitating communication in today’s appearance-conscious society. However, common issues with dentures include discomfort while chewing and dissatisfaction with esthetics and speech. Prosthetic rehabilitation for complete denture patients should aim not just at replacing missing teeth but at fully restoring masticatory functions and appearance. This article outlines a simple, economical, and esthetically pleasing approach to rehabilitating patients with complete dentures, particularly those with resorbed ridges and with difficulty in phonetics. The focus is on comprehensively restoring the patient’s orofacial complex.

## Introduction

Edentulism is a chronic condition that hinders basic functions such as eating, speaking, and socializing for those affected [1]. These challenges can lead to escalating social and psychological issues for some individuals. Additionally, tooth loss has physical repercussions, including the loss of alveolar tissue support, weakened facial muscle support, diminished biting force, etc. [2,3].

Atwood demonstrated that ridge resorption is an ongoing process following tooth extraction, leading to deteriorated jaw structure and inadequate denture support. This resorption is particularly prevalent in the lower ridges, causing significant issues with these dentures such as loss of stability and difficulty in mastication [4,5].

Dentures provide a life-like appearance in our image-conscious society, boosting patient’s confidence and facilitating social gatherings. Achieving esthetics in complete dentures involves not only the precise choice of teeth but also the restoration of lost facial anatomy, making facial esthetics a crucial component [6].

Characterized dentures have gained importance with the increased awareness and expectation of life-like replacement by the patient, and development in materials, technology, and widespread use of implants. They are designed to closely mimic the natural appearance of teeth and gums. This involves custom shading, contouring, and texturing to create a realistic look, which significantly enhances patient confidence and satisfaction. Having dentures that look and feel like natural teeth can positively impact a patient’s self-esteem and social interactions, reducing the psychological burden associated with tooth loss. Overall, characterized dentures offer a superior option for edentulous patients, combining esthetics, comfort, and functionality to improve their quality of life [7,8].

This case report outlines a simplified approach to deal with a resorbed mandibular ridge speech impairment. It involves characterizing the complete denture to suit the patient’s requirements for both a functional restoration and an esthetic enhancement. Traditional dentures support rehabilitation in these instances through a blend of different straightforward methods.

## Case presentation

**Figure 1:**
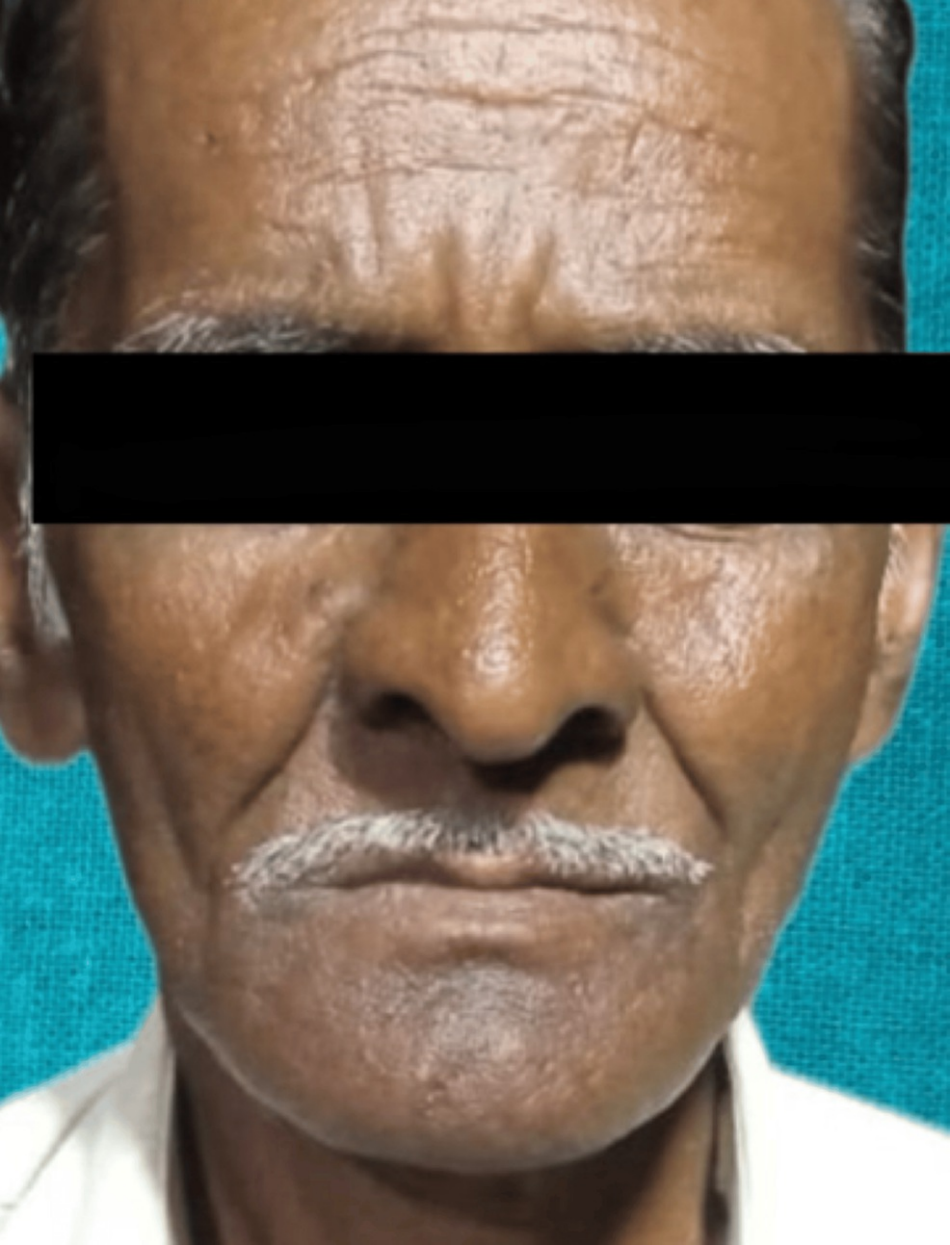
Preoperative photograph of the patient

A 76-year-old male patient who was a farmer by profession and had a 30-year habit of tobacco chewing visited the Department of Prosthetic Dentistry at Sharad Pawar Dental College and Hospital in Sawangi, Wardha. He was dissatisfied with his previous dentures because they did not provide adequate chewing quality, phonetics, and esthetics. He claimed to have problems with his chewing and speaking or pronunciation. After compiling his dental history, it was discovered that he had previously worn dentures for 6 years and had missing teeth due to periodontal disease for 10 years. The patient expressed a desire for dentures that mimic the subtle nuances of tobacco stains and natural pigmentation along with ease in pronunciation (Figure 1).

**Figure 2:**
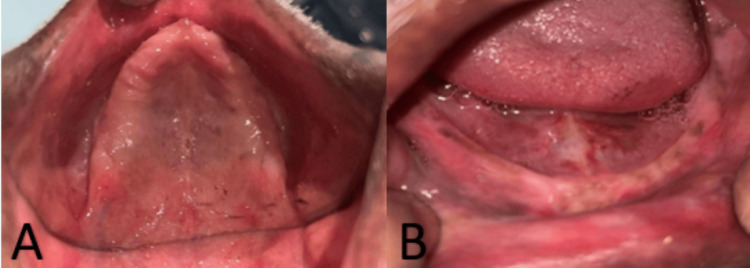
Intraoral examination of the patient - showing completely edentulous A) maxillary and B) mandibular arches

Following an intraoral examination, it was observed that the patient had complete tooth loss in both jaws, with notable ridge resorption in the lower ridge. Additional examination revealed weak and unsupported orofacial muscles. A personalized treatment plan was then devised, taking into account the patient’s specific needs and requirements. A full denture was crafted by capturing the neutral zone and positioning the teeth accordingly within this zone, while also adjusting the resorption and residual ridge impression techniques. Additionally, the denture underwent further characterization to achieve a pigmented appearance. The complete process of manufacturing the prosthesis is shown in Figure 2.

Step 1: Preliminary impressions

**Figure 3:**
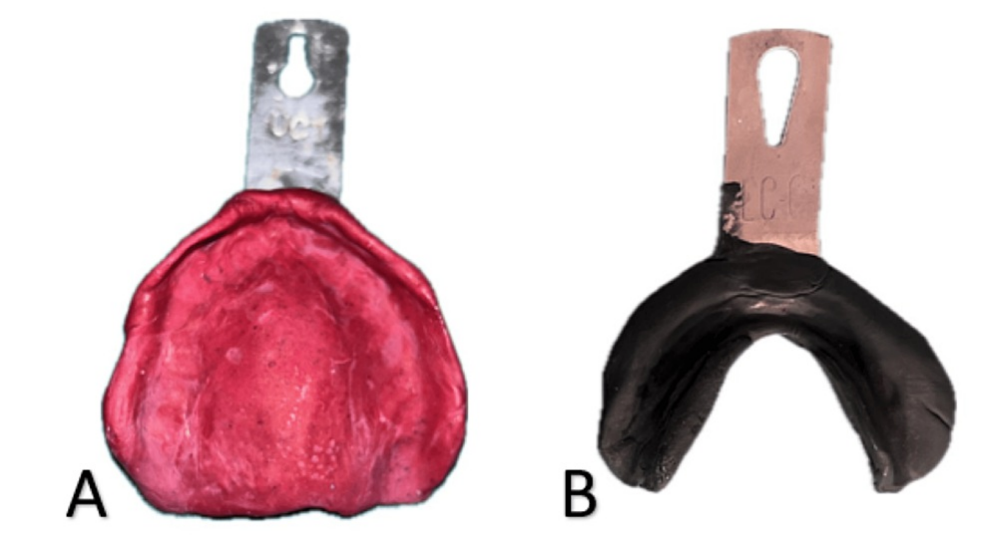
A) Maxillary and B) mandibular preliminary impressions

The maxillary preliminary impression was recorded using impression compound material and the mandibular preliminary impression was recorded with an admixed technique (3:7 ratio of impression compound and low-fusing green stick compound) (Figure 3).

Step 2: Final impressions

**Figure 4:**
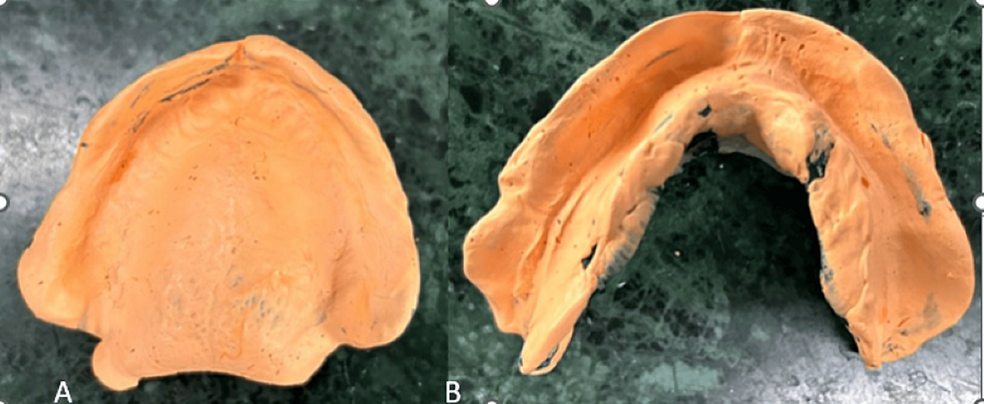
A) Maxillary and B) mandibular final impressions

The initial cast served as a foundation for fabricating a personalized tray for the border molding and final impression procedure. Border molding was done using a low-fusing green stick impression compound, followed by making final impressions using noneugenol impression paste as the patient felt a burning sensation on the palate with the eugenol impression paste. A master cast was poured using vacuum-mixed Type III dental plaster after confirming the accuracy of the final impressions (Figure 4).

Step 3: Recording of jaw relation

Temporary denture bases were created on the master cast using a self-curing acrylic material. A wax rim was employed to determine jaw relationships. After establishing the initial vertical dimension and centric relation record, a metal stopper made of stainless steel orthodontic wire was placed in the molar area. This stopper was attached to hold the cold cure material for recording the neutral zone.

A combination of impression compound and green stick material in the ratio of 3:7 was prepared and then applied to the modified mandibular denture base which was then trimmed to the desired height. Patients were instructed to perform specific movements such as swallowing, whistling, and pronouncing vowel words like a, e, i, o, u. These actions help mimic the natural movements of the muscles in the neutral zone, ensuring a more accurate fit of the denture.

**Figure 5:**
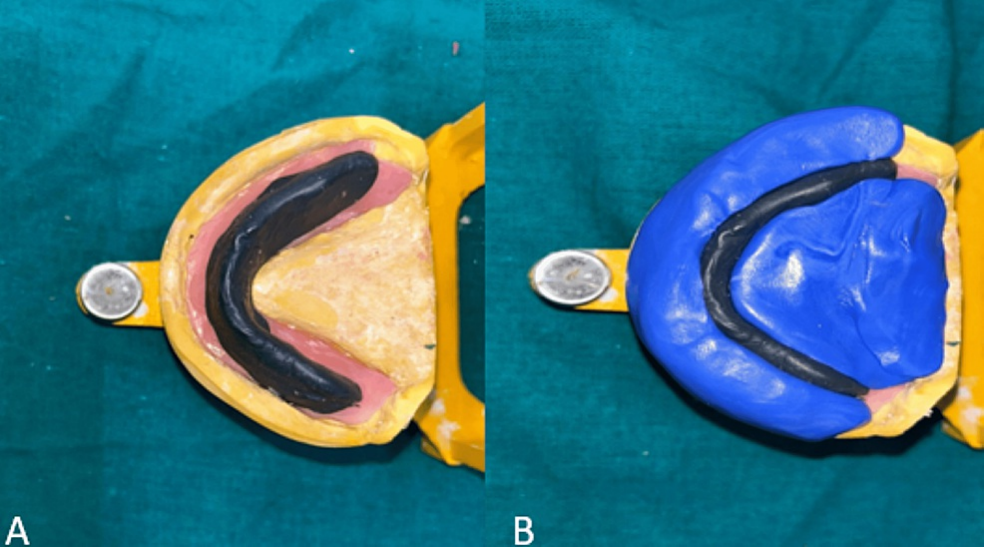
A) Neutral zone recorded, B) putty index made for the neutral zone

The mandibular denture base with the neutral zone recorded was removed. The neutral zone was maintained with a putty consistency silicone, melted modeling wax was poured between the marks, and the wax that flowed within the neutral zone formed the shape of the rim. As the wax flowed within the neutral zone, it took the precise shape required for the rim of the denture. This process helps ensure that the denture will fit comfortably and function effectively by closely matching the natural movements and contours of the patient’s mouth (Figure 5).

Step 4: Teeth arrangement and try-in procedure

**Figure 6:**
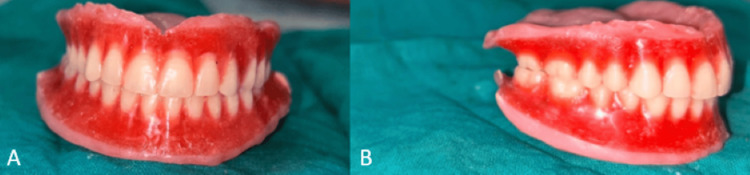
Waxed-up trial dentures

Anatomic teeth were selected for arranging the artificial teeth based on the patient’s primary complaint of difficulty in eating. The lower teeth were arranged according to the putty index, followed by the upper teeth arranged to match the mandibular setup. To maintain the contours defined by the putty indices in the neutral zone, no extra layer of wax was added to the denture flanges. The teeth were set in occlusion, and the dentures were waxed and assessed for esthetics and function during the try-in phase (Figure 6).

Step 5: Recording the palatogram

The patient was asked to pronounce specific sounds individually while keeping their mouth open so that the tongue did not touch the palate. The vowel “o” was recorded in combination with a consonant, but not as a word. For example, “ko” was used to hear the “k” sound, and similarly, combinations like “chi” and “cho” were used to prevent multiple graphic recordings.

Next, the top surface of the dentures was painted with a green food-coloring agent, chosen for its contrast with the denture base, and then the dentures were placed into the patient’s mouth, making necessary adjustments. Care was taken to ensure the painted palate did not touch anywhere else before the sounds were pronounced.

A sound palatogram was created by having the patient wipe a film of paint with their tongue, forming a pattern when speaking. These pseudo-sound palatograms were compared to reference samples of each sound. When the patient was asked to make an “S” sound, it was observed that one side of the palate had strong contact, indicating over-contouring, which was then trimmed off. Additionally, a broad area across the posterior slope of the palate also showed strong contact, suggesting excessive contouring in those regions too. The contours of the palate were precisely shaped for easy identification and correction. Overly contoured areas, particularly on the posterior slope of the palate, were then trimmed off using acrylic burs. However, when the patient was asked to produce other sounds such as alveolar, or palatal sounds, no significant markings were observed on the denture. The next step was to adjust the palatal contour to match the natural “S” curve, based on the extent of the impairment.

After inserting the denture base, the patient was asked to repeatedly pronounce “so,” “to,” and “shos” rapidly for 10 minutes to observe tongue movement during speech. Most tissue modifiers conform to the tongue to match its functional anatomy, resulting in a personalized anterior palate.

**Figure 7:**
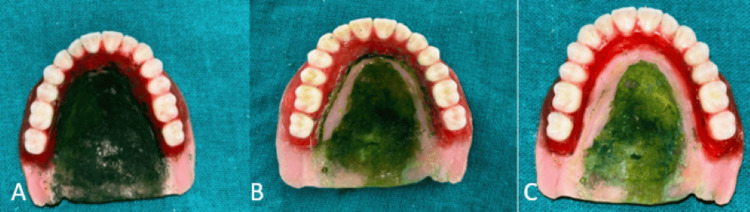
Palatogram recordings A) food-coloring agent painted over the top surface of denture, B) inappropriately contoured palate, C) properly contoured palate after selective trimming in some regions.

This process was repeated several times until the desired result was achieved. The patient was asked to repeat the test, and careful observation confirmed reliable palatal contact. The patient was instructed not to intentionally touch the palate with the tongue during the palatogram recording. Once the clarity of sibilance was confirmed, the prosthesis was removed from the patient’s mouth without disturbing the recorded palatal images (Figure 7).

Step 5: Flasking, dewaxing, and packing procedure

**Figure 8:**
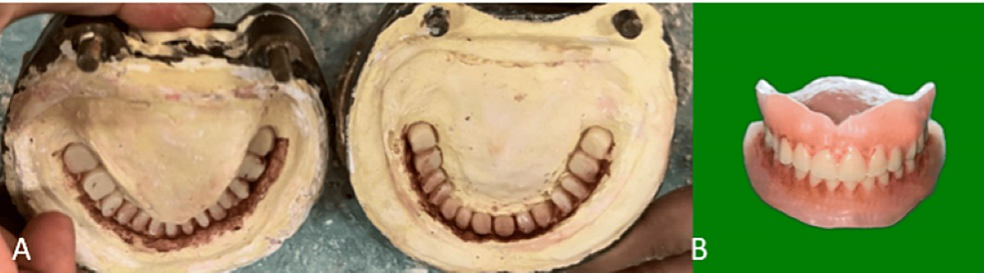
A) Extrinsic stains painted on the dental stone, B) stains incorporated in the final dentures

During this procedure, the characterization of dentures was performed to replicate the tobacco stains and pigmentation by giving them a more natural appearance. Extrinsic stains were mixed with heat-cure monomer to ensure proper incorporation of stains into the denture and were applied on the dental stone after the process of flasking and dewaxing as shown in Figure 8.

**Figure 9:**
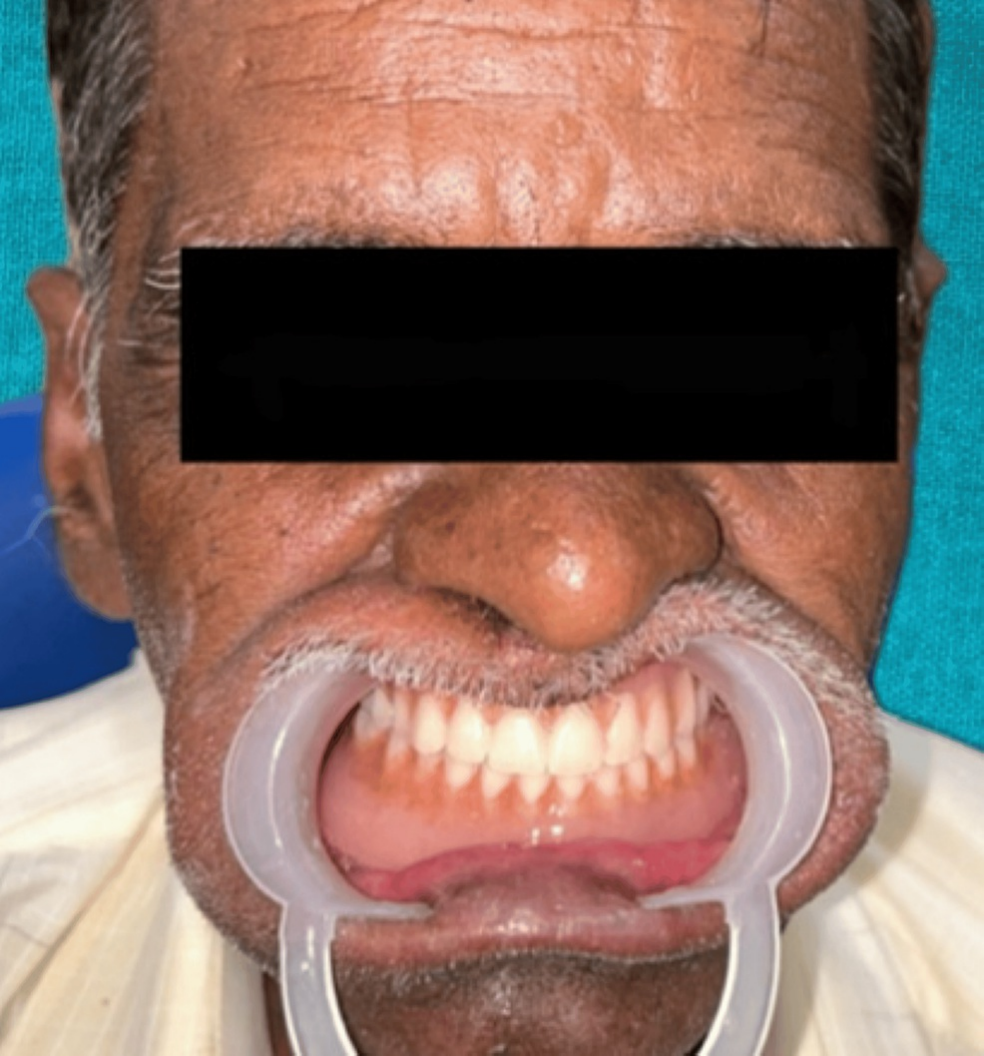
Postoperative photograph

The patient was then recalled 24 hours and 7 days post-insertion. After 24 hours, the patient was satisfied with the complete dentures, requiring only minor adjustments. Once these issues were addressed, the patient was recalled 7 days later. Regular follow-up visits were scheduled to assess function, esthetics, and retention (Figure 9).

## Discussion

Tooth loss is likely to remain a prevalent issue in the foreseeable future, leading to the need for prosthetic replacements. Complete dentures can help restore esthetics and function to some extent. Although many edentulous patients adjust effectively to their condition and prostheses, some encounter notable functional and psychological difficulties. While the use of anatomical teeth for resorbed contours is a topic of debate, arranging anatomical teeth in a balanced occlusal pattern might enhance chewing efficiency for patients [8].

The neutral zone technique is a critical approach in the fabrication of dentures, particularly for patients with complex dental needs or compromised oral anatomy. This technique involves identifying and recording the area where the forces of the tongue, lips, and cheeks are in equilibrium, ensuring the optimal placement of artificial teeth and denture bases. This technique takes into account the individual characteristics of the patient’s oral musculature, resulting in a prosthesis that is tailored to their specific needs, enhancing satisfaction, and compliance [9].

Tanaka studied how palatal contour affects speech intelligibility. He observed that the anterior palate has a reverse curve in a sagittal section, which is essential for pronouncing “S” and “SH” sounds. When comparing the palatal contours of dentulous patients to those with maxillary dentures, he found that most dentures lack this crucial feature. An alveolopalatal prominence should start at the premolar teeth and thicken toward the molars, enabling the tongue to seal the posterior palatal area of a denture and direct the airflow toward the anterior region. However, many dentures have a concavity instead of this prominence, leading to phonetic issues [10].

Allen noted that the common practice of randomly thinning the maxillary palatal surface to create adequate space for the placement of the tongue often neglects the importance of correct palatal contours for sound production. Changes in palatal contours with new maxillary dentures can cause phonetic alterations, particularly affecting the “S” sound, which might manifest as a “whistling S” or an “Sh” sound due to improper airflow. Correct formation of the “S” sound requires a correctly sized medial groove in the tongue. Other palatolingual consonants like “T,” “D,” “N,” and “L” are also impacted by changes in palatal contours. Excessive thickness in the anterior palate can cause premature contact, resulting in the “T” sounding like a “D.” The replication of rugae in dentures is debated; some believe they are necessary for proper sound formation, while others think they add unnecessary thickness. Allen suggested increasing the thickness of the denture around the region of the incisive papilla to control airflow during the pronunciation of “S” [10,11].

Denture staining is a critical component of prosthetic dentistry focused on improving the visual appeal of dentures by imitating the authentic features of teeth and gums. This procedure includes applying stains to the denture material to recreate the natural color tones and textures seen in real teeth and gingiva [12,13].

Dentures that look natural can significantly boost a patient’s self-confidence and self-worth, leading to better social interactions and an improved quality of life. Tailoring the color and texture through staining enables practitioners to fulfill each patient’s specific needs and desires, creating customized prostheses that match their esthetic preferences. In older patients, staining can replicate natural age-related changes such as subtle discoloration, enhancing the realism and acceptance of the dentures [14,15].

A mixture of impression compound and green stick material was applied to a trimmed mandibular denture base, and the patient performed various mouth movements to mimic natural muscle actions for a precise fit. The neutral zone was recorded with silicone, and melted modeling wax was used to create the occlusal rim within this zone. This process ensures a comfortable and functional denture that closely matches the patient’s mouth movements and contours which would further help to retain the mandibular denture. A sound palatogram technique, involving tongue contact patterns with a film of paint, was used to adjust the denture base. Self-curing acrylic resins were chosen for their ease of use, cost-effectiveness, and stability. Green food-coloring agent provided strong contrast for recording materials and was safe if swallowed. Adjustments were made by comparing paint patterns to reference samples, ensuring the final palatal contours allowed for clear pronunciation and speech clarity.

## Conclusions

The case report presents a straightforward and unique method for fabricating dental prostheses. While palatograms are commonly used, this technique is notably easy to implement. It effectively addresses issues related to residual ridge resorption in the mandibular arch, achieving both form and function.

Deviations from natural speech immediately after complete denture rehabilitation are normal, and a pretreatment speech diagnosis is essential for accurate comparison. Helping patients adapt to these speech changes can improve their overall adaptability. Palatograms are valuable for identifying these deviations and confirming corrected palatal contours. This simple, low-cost technique is ideal for patients with full dentures and speech disorders.

The neutral zone technique is crucial for making complete dentures that are stable, comfortable, and functional. It captures the balanced position of tongue and cheek forces, reducing denture movement during daily activities like speaking, chewing, and swallowing. This technique improves speech clarity and chewing effectiveness by aligning dentures with the dynamic movements of mouth muscles. It also enhances denture esthetics by supporting facial muscles, preventing a sunken appearance. Overall, the neutral zone technique is essential for ensuring patient satisfaction and good oral health, making it a vital part of prosthodontics.
